# Supplementary material for: Optimizing the Methodology for Antioxidant Activity Analysis of Manuka Honey
Source: Foods. 2025 Apr 14;14(8):1341. doi: 10.3390/foods14081341 (PMC12027450; doi:10.3390/foods14081341)
Supplement: Supplementary file 1 [file foods-14-01341-s001.zip › foods-3526502-supplementary.pdf]

Table S1. The intra-day and inter-day precision of different honey and de-sugared honey under various antioxidant methods.

Sample	Methods	Quercetin Equivalent (IC_{50}-based) mg/100g									Intra-RSD(%)	Inter-RSD(%)
A	ABTS	26.04	26.15	26.52	26.04	26.15	26.52	26.23	26.23	26.3	0.96	0.68
	DPPH	17.15	17.28	17.81	17.47	17.15	17.28	17.71	17.57	17.2	2.01	1.42
	CAA（Caco-2）	4.12	3.8	4.24	3.55	3.35	3.22	3.39	3.53	3.59	5.61	9.52
	CAA（HepG2）	5.33	5.65	5.87	5.89	5.25	6.32	6.04	5.23	6.18	4.83	7.10
B	ABTS	24.02	24.48	24.3	24.56	24.63	24.68	24.84	25.07	24.84	0.96	1.27
	DPPH	18.99	19.42	19.05	20.74	20.75	19.96	19.68	19.92	19.94	1.22	3.20
	CAA（Caco-2）	4.31	4.41	3.93	3.57	3.62	3.81	3.92	4.46	4.56	6.01	9.23
	CAA（HepG2）	5.79	5.54	6.07	6.01	6.51	6.78	5.79	5.65	5.43	4.57	7.51
C	ABTS	26	25.93	26.18	26.16	26.5	26.54	26.62	26.69	26.81	0.50	1.21
	DPPH	17.41	17.03	17.45	17.13	17.3	17.76	17.54	18.41	17.74	1.34	2.35
	CAA（Caco-2）	5.14	5.26	5.65	5.26	6.11	5.66	6.48	6.17	6.41	4.98	8.90
	CAA（HepG2）	7.29	7.52	7.06	6.27	6.68	6.34	6.52	6.92	6.64	3.16	6.26
Without sugar A	CAA（Caco-2）	12.53	12.07	11.79	11.43	11.95	11.64	12.56	12.91	12.74	3.08	4.29
	CAA（HepG2）	25.48	25.72	26.87	25.35	25.61	26.99	24.21	24.69	24.97	2.86	3.60
Without sugar B	CAA（Caco-2）	14.41	14.77	15.36	15.72	15.57	15.8	16.85	16.1	15.95	3.23	4.61
	CAA（HepG2）	24.29	24.66	23.35	24.76	23.34	23.92	25.46	25.3	25.63	2.80	3.52
Without sugar C	CAA（Caco-2）	14.09	14.63	14.87	16.65	16.52	16.19	14.96	15.22	16.35	2.75	6.07
	CAA（HepG2）	25.23	25.53	24.4	24.19	26.26	26.34	26.39	26.52	25.23	2.34	3.43
